# A Transgender Chatbot (Amanda Selfie) to Create Pre-exposure Prophylaxis Demand Among Adolescents in Brazil: Assessment of Acceptability, Functionality, Usability, and Results

**DOI:** 10.2196/41881

**Published:** 2023-06-23

**Authors:** Paula Massa, Dulce Aurélia de Souza Ferraz, Laio Magno, Ana Paula Silva, Marília Greco, Inês Dourado, Alexandre Grangeiro

**Affiliations:** 1 Faculdade de Medicina Preventiva Universidade de São Paulo São Paulo Brazil; 2 Unité Mixte de Recherche 1296 Radiations: défense, santé et environnements Lyon 2 University Lyon France; 3 Escola de Governo em Saúde Gerencia Regional Brasília Fundação Oswaldo Cruz Brasília Brazil; 4 Instituto de Saúde Coletiva Universidade Federal da Bahia Salvador Brazil; 5 Departamento de Ciências da Vida Universidade do Estado da Bahia Salvador Brazil; 6 Faculdade de Medicina Universidade Federal de Minas Gerais Belo Horizonte Brazil

**Keywords:** artificial intelligence, adolescent, HIV pre-exposure prophylaxis, transgender women, men who have sex with men, chatbot, PrEP, transgender, HIV, prevention, prophylaxis, acceptability

## Abstract

**Background:**

HIV incidence rates have increased in adolescent men who have sex with men (AMSM) and adolescent transgender women (ATGW). Thus, it is essential to promote access to HIV prevention, including pre-exposure prophylaxis (PrEP), among these groups. Moreover, using artificial intelligence and online social platforms to create demand and access to health care services are essential tools for adolescents and youth.

**Objective:**

This study aims to describe the participative process of developing a chatbot using artificial intelligence to create demand for PrEP use among AMSM and ATGW in Brazil. Furthermore, it analyzes the chatbot’s acceptability, functionality, and usability and its results on the demand creation for PrEP.

**Methods:**

The chatbot Amanda Selfie integrates the demand creation strategies based on social networks (DCSSNs) of the PrEP1519 study. She was conceived as a Black transgender woman and to function as a virtual peer educator. The development process occurred in 3 phases (conception, trial, and final version) and lasted 21 months. A mixed methodology was used for the evaluations. Qualitative approaches, such as in-depth adolescent interviews, were used to analyze acceptability and usability, while quantitative methods were used to analyze the functionality and result of the demand creation for PrEP based on interactions with Amanda and information from health care services about using PrEP. To evaluate Amanda’s result on the demand creation for PrEP, we analyzed sociodemographic profiles of adolescents who interacted at least once with her and developed a cascade model containing the number of people at various stages between the first interaction and initiation of PrEP (PrEP uptake). These indicators were compared with other DCSs developed in the PrEP1519 study using chi-square tests and residual analysis (*P*=.05).

**Results:**

Amanda Selfie was well accepted as a peer educator, clearly and objectively communicating on topics such as gender identity, sexual experiences, HIV, and PrEP. The chatbot proved appropriate for answering questions in an agile and confidential manner, using the language used by AMSM and ATGW and with a greater sense of security and less judgment. The interactions with Amanda Selfie combined with a health professional were well evaluated and improved the appointment scheduling. The chatbot interacted with most people (757/1239, 61.1%) reached by the DCSSNs. However, when compared with the other DCSSNs, Amanda was not efficient in identifying AMSM/ATGW (359/482, 74.5% vs 130/757, 17.2% of total interactions, respectively) and in PrEP uptake (90/359, 25.1% vs 19/130, 14.6%). The following profiles were associated (*P*<.001) with Amanda Selfie’s demand creation, when compared with other DCS: ATGW and adolescents with higher levels of schooling and White skin color.

**Conclusions:**

Using a chatbot to create PrEP demand among AMSM and ATGW was well accepted, especially for ATGW with higher levels of schooling. A complimentary dialog with a health professional increased PrEP uptake, although it remained lower than the results of the other DCSSNs.

## Introduction

In Brazil, the HIV epidemic has disproportionately affected young people, especially men who have sex with men (MSM) and transgender women (TGW) [1,2]. This trend is explained by a worsening response to HIV in recent years [3] and behavioral changes in younger generations, marked by increased unprotected anal intercourse, reduced HIV risk perception, and less access to HIV/AIDS information [4].

In addition, adolescent men who have sex with men (AMSM) have less knowledge and are less willing to use new HIV preventive methods, such as pre-exposure prophylaxis (PrEP), compared with older MSM [5]. This reality has demonstrated the importance of developing HIV prevention strategies that are more consistent with the values and practices of younger generations, emphasizing the use of digital technologies and social media [6-8]. Such technologies include chatbots, which are increasingly used in the health care system for different purposes, such as improving access to information, supporting decision-making, creating demand strategies, and addressing specific topics (eg, physical activity, diet, adherence, and treatment of diseases) [9]. Interaction with users employs computational techniques (artificial intelligence) to identify an individual’s intention through keywords and select standardized responses in a database.

The acceptability of chatbots has been correlated with the offer of easy interactions focused on the main content through the use of human conversation vocabulary; the chatbot’s ability to motivate the user, who should feel in control of the interaction; and its efficiency in providing assistance and information [10]. By contrast, when emotional support is intrinsic to the topic of the chat, it is not recommended to use cognitive empathy, defined as the comprehension of another person’s emotions, because it might be perceived as awkward [11].

Chatbots’ advantages in health care are reaching people with low access to health care services, low cost, permanent availability, and greater convenience [12]. Chatbots have also been viewed as tools for improving the relationship between users, institutions, and health care professionals by addressing sensitive issues without direct human interaction. Moreover, it contributes to the users’ impression of being shielded from prejudgments. By contrast, users may need clarification about the accuracy and safety of information provided by chatbots precisely because they are not humans [13,14]. However, compared with other digital tools, such as apps for health monitoring, chatbots may contribute to improving users’ satisfaction and retention. They can be particularly beneficial for users with a poor attitude toward using technology, helping them to find out the information they need, which can contribute to reducing the digital divide and to improving social inclusion [15].

In the field of HIV, positive experiences have been reported, with chatbots providing flexibility and agility in contacting health teams and institutions [16], as well as introducing counseling to young people quickly and reliably [17]. By contrast, chatbots sending reminders for medication use and scheduling consultation can be perceived as invasive and less accepted [16]. To the best of our knowledge, there are no published studies on the ability of chatbots to disseminate information and create demand for PrEP, especially for AMSM and adolescent transgender women (ATGW). Aiming to contribute to filling this knowledge gap, we herein describe the participative process of developing a chatbot to create demand for PrEP use among adolescent MSM and TGW in Brazil and analyzed its acceptability, functionality, usability, and results.

## Methods

### Chatbot Proposition: Amanda Selfie

Amanda Selfie was created within the PrEP1519 study as one of the strategies for creating demand for PrEP use among AMSM and ATGW. PrEP1519 is a demonstration study on the effectiveness of daily PrEP among AMSM/ATGW aged 15-19, carried out in 3 Brazilian capital cities: Salvador, Belo Horizonte, and São Paulo. The eligibility criteria were HIV negative and at a greater risk of HIV [18]. At enrollment, participants can choose to use PrEP (comprising the PrEP arm) or other prevention methods delivered by the study (comprising the non-PrEP arm). Besides the chatbot, peer educators implemented other similar demand-creation strategies based on social networks (DCSSN) on Instagram (Meta Platforms, Inc.), Facebook (Meta Platforms, Inc.), and WhatsApp (Meta Platforms, Inc.). More details about these and the whole demand creation strategies (DCSs), implemented in person and through relationship apps, can be found in Magno et al [19].

Demand creation is defined as the improvement of knowledge, attitude, motivation, intention, decision-making, and need for health care services to initiate the use of PrEP, among the AMSM and ATGW population. For this purpose, in the PrEP1519 study, demand creation included activities that extend from identifying people who can be benefited by PrEP, as they are at a greater risk of HIV infection, until providing information and counseling to overcoming barriers to starting the use of the prophylaxis in the health service (PrEP uptake).

Amanda Selfie was developed to provide information on sexual health and gender identity issues; to identify, through an online questionnaire, AMSM and ATGW at a greater risk of HIV infection; to offer access to the scheduling of appointments for PrEP enrollment as part of the PrEP1519 study; and to improve linking and to retain adolescents in the PrEP clinics by identifying risk situations with the need for clinical care for sexually transmitted infections (STIs) and postexposure prophylaxis, making appointments at the request of the users, sending reminders to take the PrEP pills, and offering HIV self-tests that were mailed (postal) to participants.

### Development Plan

#### Overview of Stages

The chatbot was developed for the Facebook Messenger platform (Meta Platforms, Inc.) [20]. The artificial intelligence engine used was Dialogflow (Google LLC/Alphabet Inc.). It used the Perl (Larry Wall/The Perl Foundation) and JavaScript (Brendan Eich/Oracle Corporation) programming languages, with the Bottender framework [21], and communicated with an application programming interface in Perl using the Catalyst [22] and Mojo [23] frameworks. Facebook Messenger was chosen because it is the one that allows the use of more elaborate conversation flows and at the same time offers free mobile data packages in Brazil. However, this option required more effort to disseminate the chatbot (strategies described in the “Stage 1: Conception” section) because adolescents have migrated to other messaging platforms more recently.

The chatbot transfers to the PrEP1519 database selected information (eg, demographics characteristics and increased risk for HIV) actively obtained during user interactions on closed flows. The transfers occur when the chatbot identifies the user as AMSM or ATGW. The selected data were registered in the database through a web service developed in the PHP (Hypertext Preprocessor) programming language (PHP Group). The PrEP1519 team used this information to improve management and clinical care and used it only with electronic consent given by the adolescent.

**Figure 1:**
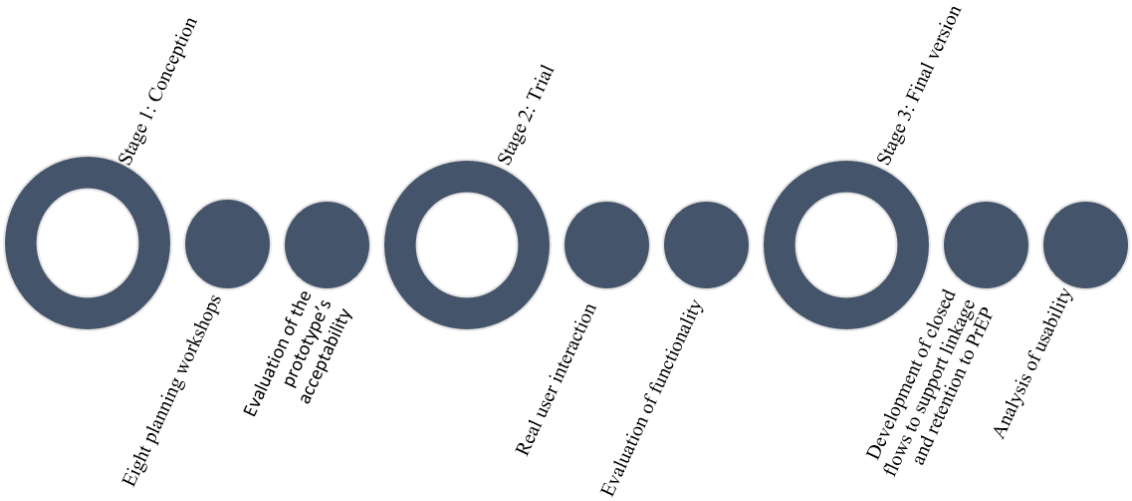
Stages of Amanda Selfie's participative development. PrEP: pre-exposure prophylaxis.

The development of Amanda Selfie lasted from October 2018 to June 2020 and comprised 3 stages: conception (October 2018 to June 2019), trial (June to December 2019), and final version, after revision and adjustments (January to June 2020; Figure 1). A formative evaluation accompanied the entire process.

#### Stage 1: Conception

**Figure 2:**
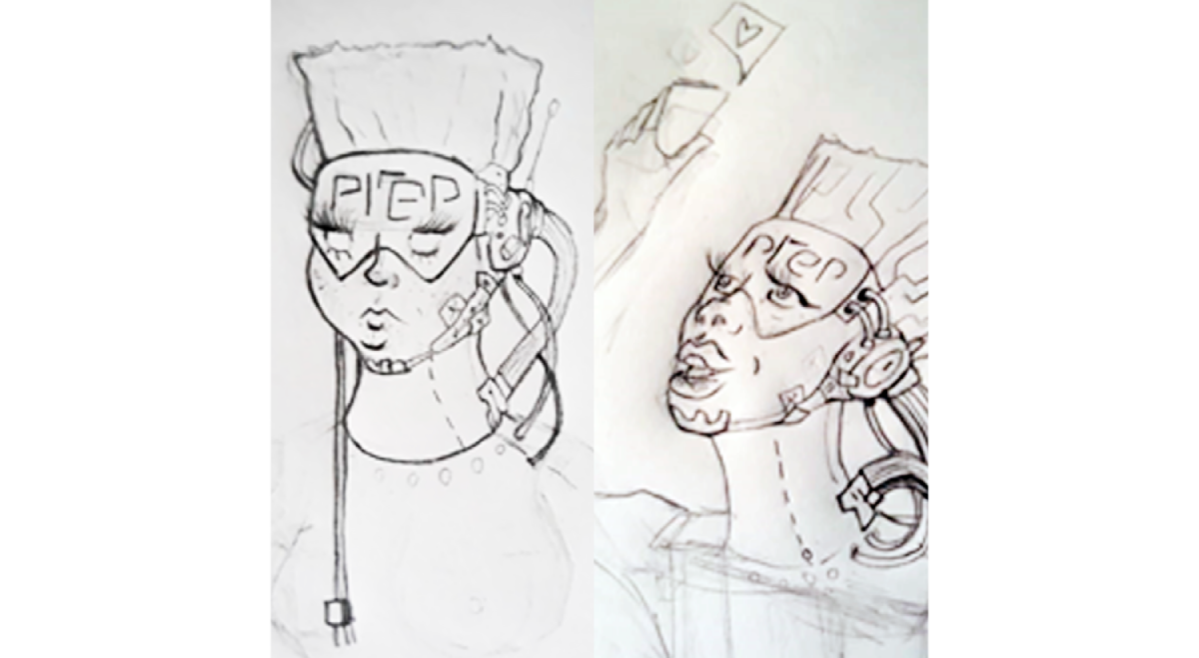
Illustrations of Amanda Selfie’s prototype persona during the development process. PrEP: pre-exposure prophylaxis.

The first stage was the conception of the persona, its image (Figure 2), and language; the definition of objectives and the initial proposal of open conversation flow and closed flow for demand creation; the building of an interaction monitoring panel; and the integration with the PrEP1519 database. This stage took place in 8 planning workshops with the participation of professionals and researchers working in the fields of communication, health, and information technology, as well as AMSM and ATGW.

When developing the open flow, questions initially identified as relevant by health care professionals were inserted based on what they commonly encountered while providing PrEP to young people. The following themes were prioritized: HIV, sexual health, and gender identity.

**Figure 3:**
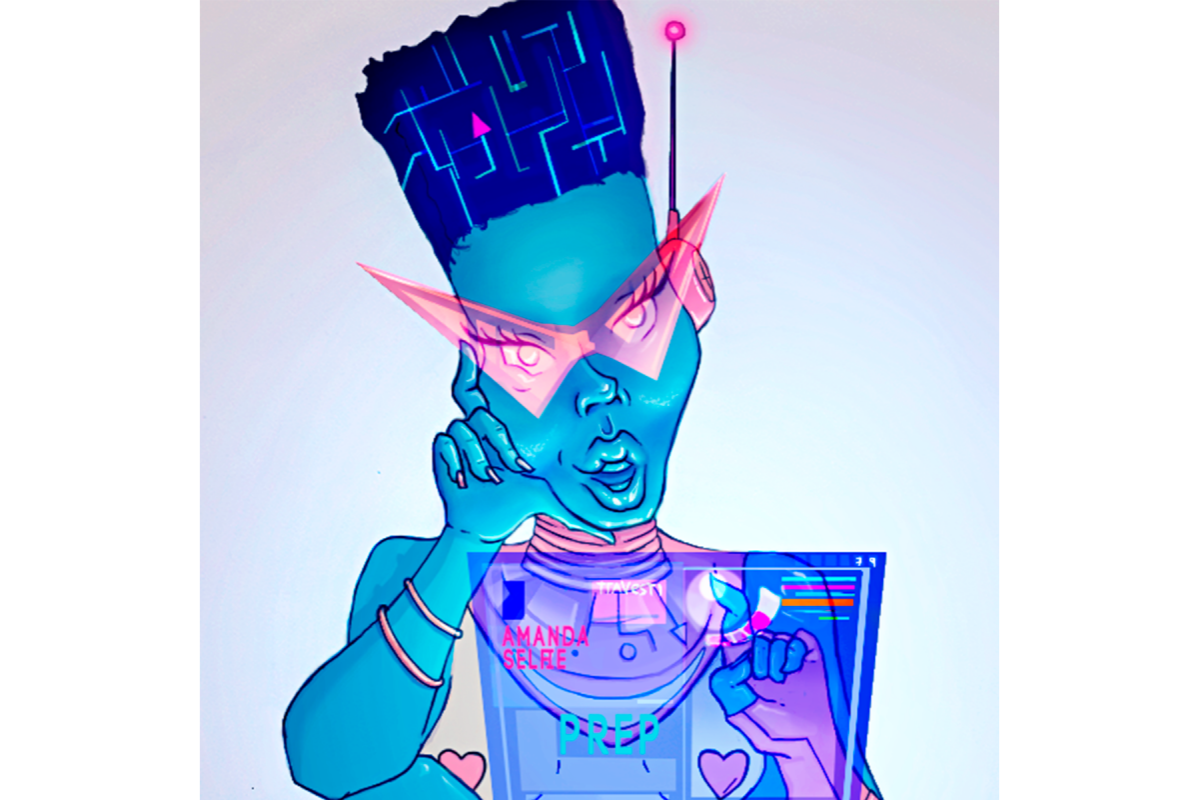
First image of Amanda Selfie. PrEP: pre-exposure prophylaxis.

The first evaluation of the prototype’s acceptability occurred during this stage. To this end, we conducted 9 in-depth individual interviews with adolescents selected by partner nongovernmental organizations and 2 discussion groups with 6 peer educators each. An interview script was used to explore participants’ perception of the chatbot’s image, their confidence to interact with it, acceptance of being part of a health service, and empathy with the adolescent audience. The first image (Figure 3)—the prototype’s characteristics—and the chatbot’s objectives were presented before the interviews.

The results improved the prototype and developed the first open and closed flows. The open flow was formed by questions typed freely by the user that triggered Amanda Selfie’s answers extracted from the database. The closed flow was formed by questions asked by Amanda Selfie, with user responses given through standardized buttons. Before the trial stage, Amanda Selfie was launched to the public using 3 strategies: (1) making and promoting posts on Facebook and Instagram; (2) distributing printed material in AMSM and ATGW social gatherings and health care services; and (3) disseminating short videos using WhatsApp and other social media to PrEP1519 participants and contacts.

#### Stage 2: Trial

The trial phase aimed to initiate real user interaction with Amanda to improve the accuracy of open and closed flows for demand creation. For this purpose, all interactions were assessed by a team of health professionals and peer educators, who analyzed the consistency and accuracy of Amanda Selfie’s responses. Any questions initially unforeseen or insufficiently answered by Amanda in the interactions with real users were reviewed to develop new content on the platform or improve the existing text.

Before any inclusion on the platform, the health care professional’s text was rewritten by a TGW writer and a group of AMSM and ATGW PrEP1519 participants to establish a better relationship between the Amanda Selfie persona, her responses, and the adolescent audience. When an open or closed flow did not work correctly, and this error could lead to any harm to the user, a team member contacted the user to understand the demand and establish appropriate referrals. This analysis also helped to improve the functioning of the chatbot.

Additionally, an assessment of the functionality of open and closed flows was performed using the indicator panel exclusively designed for the chatbot. The panel systematizes information about the total number of users by age group, the number of users who started and how many of them completed each interaction stage, and the number of times each topic of interest was accessed.

Thus, the indicator panel made it possible to identify closed demand creation flows that had low rates of completeness and, in open flows, the themes most demanded by adolescents based on the frequency with which a user comment or question triggered a topic from Amanda Selfie’s response database. The results of these assessments were used to refine the response database, access keys, and flow steps in the third stage.

#### Stage 3: Final version

The third and final stage consisted of developing closed flows to support linkage and retention to use PrEP and the usability evaluation of all of Amanda’s closed flows.

A total of 18 in-depth interviews were conducted with adolescents contacted by the project team for the qualitative usability evaluation; 6 of them had enrolled in the PrEP arm, 6 in the non-PrEP arm, and 6 had been reached by the study DCSSN but did not enroll. Selection criteria for each group included at least 3 participants between 15 and 17 years old, 3 TGW, and 3 MSM. The interviews were conducted remotely using WhatsApp. Before the interviews, participants received a video encouraging the use of Amanda Selfie and explaining how to start using the chatbot. The interviews were guided by an interview script comprising perceptions of Amanda Selfie’s ability to communicate; the agility, objectivity, and accuracy of answers; providing comfort and confidence in establishing interactions; ensuring completeness of the topics addressed; and suggestions for changes.

### Evaluation of Amanda’s Result of Demand Creation for PrEP

After the third stage and the final version of the chatbot, we analyzed Amanda’s ability to create PrEP demand as follows: quantifying the number of AMSM and ATGW at an increased risk for HIV who interacted with Amanda for the first time; the sociodemographic characteristics of these adolescents; and the frequency of PrEP uptake after this interaction. For analysis, we constructed a cascade model with the number of people who initiated and completed the following steps: initial interaction, scheduling at the service, and PrEP uptake. To deepen our understanding of Amanda’s contribution to creating demand, we compared the sociodemographic characteristics (gender, age, skin color, and education level) of adolescents she approached with other DCSSN and face-to-face strategies. This analysis used the data collected between June 2019 and March 2021, and chi-square tests and standardized residue with a significance level (*P* value) of .05 were used to study associations.

### Ethical Aspects

The PrEP1519 study was performed according to the Brazilian (Resolution CNS no. 466, Brazil, 2012) and international research ethics guidelines. It was approved by the Research Ethics Committees of the World Health Organization, Federal University of Bahia-UFBA (approval number 3,224,384), University of São Paulo (approval number 3,082,360), Federal University of Minas Gerais (approval number 2,027,889), and Federal University of Bahia (approval number 3,224,384). All participants could withdraw consent at any stage of the process or skip any questions perceived as too sensitive, too personal, or distressing. To guarantee confidentiality, all data were stored in a special and safe database, and no personal information was used for any public presentation or publication.

## Results

### Overview

The presentation of the results was organized according to the stages of development. We describe each version of Amanda in the respective stage. Then we present the results of qualitative and quantitative analyses that supported the decisions for development.

### Conception Version

Amanda Selfie’s persona was conceived as a young TGW with futuristic clothes and accessories, and her image had the facial features of a black woman and blue skin color. The choice of a persona representing a more vulnerable and stigmatized social group in Brazilian culture aimed to generate feelings of empathy for individuals who often have less access to health care services and to promote the positive affirmation of TGW. We also assumed that women with a transgender persona would communicate better with the diversity of the groups than cisgender men. The futuristic attire and the blue color marked the robotic nature and reference to the PrEP pill, and the African ancestry refers to Brazilianness. The setting in a health care service sought to reinforce the welcoming role of the chatbot.

For written communication, the Pajubá language was used as a reference. Pajubá is a linguistic-cultural instrument that aggregates African vocabulary—especially elements from the Yoruba and Nago languages—to that of the Brazilian TGW community. Pajubá is a language developed as a form of resistance to their condition of marginalization and, over time, gained popularity in the lesbian, gay, bisexual, transgender, queen, intersexual Brazilian community [24]. Gender-neutral language was also adopted, ensuring the inclusion of nonbinary people.

The closed flow, which aimed to create demand for PrEP use, was composed of a single flow with 3 stages: (1) responding to a quiz called “Who are you in the groove?”, which aimed to make the interaction more fun by offering “the profile” of the user in social interaction and flirting spaces; (2) identifying AMSM and ATGW and characterizing them in terms of situations of sexual risk for HIV; and (3) offering to schedule an appointment at the PrEP clinics for those eligible for PrEP. Before scheduling an appointment, Amanda Selfie explained the objectives of the PrEP1519 study and the users’ rights and requested consent to use data collected in the dialogs for research purposes.

In the prototype’s acceptability evaluation, adolescents suggested that, for a greater acceptance of Amanda Selfie, a “balanced persona” should be adopted—one that emphasized her identity and affirmed her gender diversity, albeit without becoming caricatured or exaggerated. On the one hand, the attributes highlighted by the audience of interest (eg, African ancestry traits, age group, and gender) increased acceptance. On the other hand, the marked sensuality of her facial expressions and clothing and being a virtual assistant caused some strangeness.

**Figure 4:**
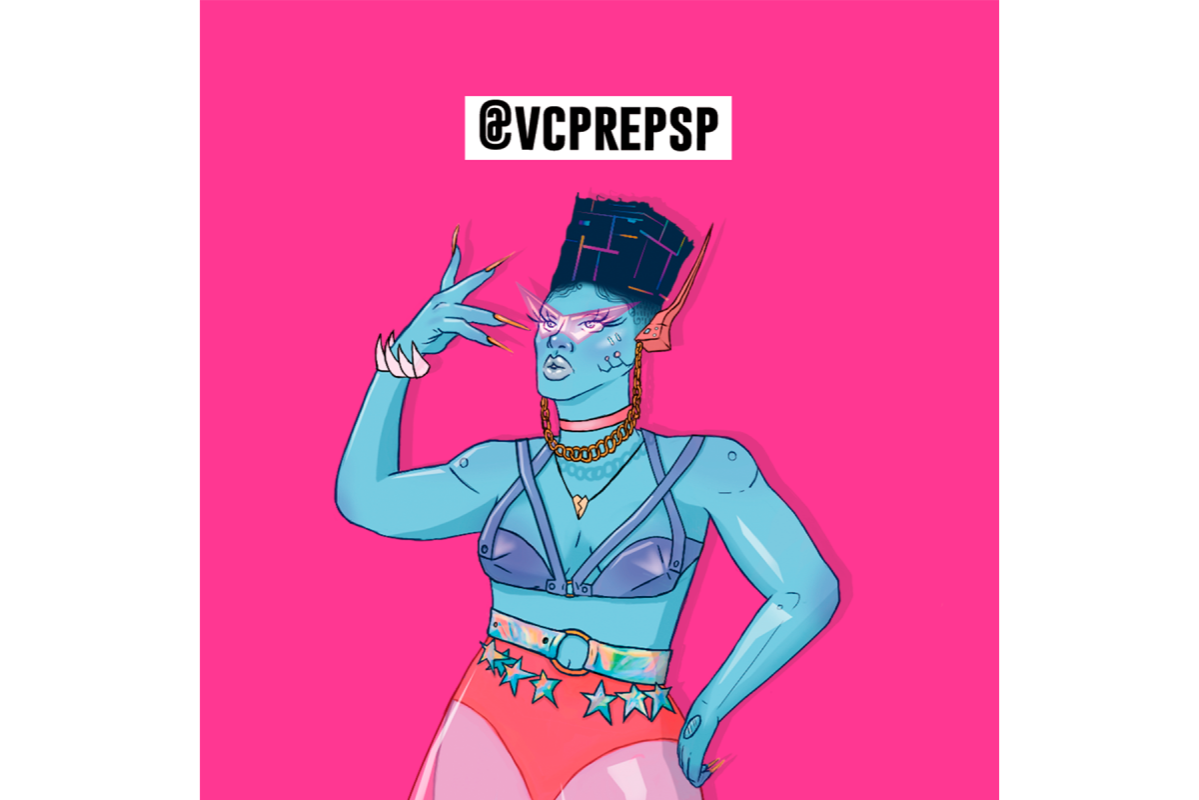
Final image of Amanda Selfie.

Regarding her appearance, most interviewees considered Amanda Selfie to be “different,” “futuristic,” and “humanoid”—characteristics that were positively evaluated. However, 3 adolescents expressed strangeness with the character, criticizing the blue color (no one is blue) and suggesting that her African features should be reinforced. They also reported difficulty recognizing a TGW robot in the illustration and considered her shy and inexpressive. Such considerations led to a revision of Amanda Selfie, which resulted in the final image of the chatbot (Figure 4).

### Trial Version

At the end of the trial stage, the closed flow for demand creation was simplified, making gender and age questions mandatory to identify whether the participants are AMSM or ATGW. The questions on HIV risks became optional, and the quiz (stage A of the first version) was excluded for adolescent users. This simplification facilitated scheduling appointments immediately after the initial questions. Flows for human interaction were included, and users could choose how they prefer to continue the conversation: in person or remotely, as well as through WhatsApp or Instagram. These changes resulted from functionality evaluations and led to a shift from a single flow with 3 stages to 7 closed demand creation flows to be used based on the interaction choices made by adolescents.

The functionality evaluation revealed how a small proportion of people who went through the entire flow and reached the moment of being offered appointments for PrEP. In this flow, 41.0% (50/122) of adolescents interrupted the interaction at the quiz or identification of HIV risk. Among those who reported sexual risk, 33% (23/69) interrupted the interaction when asked about their interest in scheduling an appointment, whereas 17% (12/69) scheduled and 13% (9/69) attended an initial appointment.

These aspects indicated that including a quiz at the beginning of the flow could be acting more as a barrier than as motivation to use the chatbot or enroll in PrEP. Furthermore, a completely closed flow to encourage PrEP enrollment proved inadequate for adolescents who needed more information, more time to decide, or both. Consequently, after the new flow, which was more objective and more directed toward PrEP, was implemented, we observed an increase in appointment scheduling from 17% (12/69) to 46% (26/57).

Regarding the open stream, Amanda’s final version has a database with 119 themes (Multimedia Appendix 1). The evaluation of the open flows indicated that the topics most frequently asked by users were information about HIV (477/757, 63%) and new preventive methods, especially PrEP (326/757, 43.1%), followed by topics such as sexual experiences, sexual orientation, and gender identity (280/757, 37%).

### Final Version of Amanda

The final version of Amanda Selfie has a total of 26 closed flows, which are available according to the type of audience (eg, adolescents, PrEP users) and purpose of the interaction (eg, information, demand for health care). These flows are organized into 2 groups: (1) PrEP demand creation, which involved identifying people at risk of HIV and offering PrEP (Multimedia Appendix 2); and (2) linkage and retention of adolescents using PrEP or other preventive methods. The second group of flows is new in this version.

The 19 closed flows of linkage and retention to care strategies are aimed exclusively at adolescents using PrEP. Amanda Selfie recognized these adolescents from their institutional link and PrEP use situation (eg, the PrEP1519 study, the PrEP-Brazilian National Health System), which allows the chatbot to offer personalized flows. The offer of consultations occurs, preferably through a menu called “things went bad.” In this menu, Amanda Selfie uses questions and standardized answers to identify situations requiring health care because of a higher risk of exposure to HIV and other STIs.

**Figure 5:**
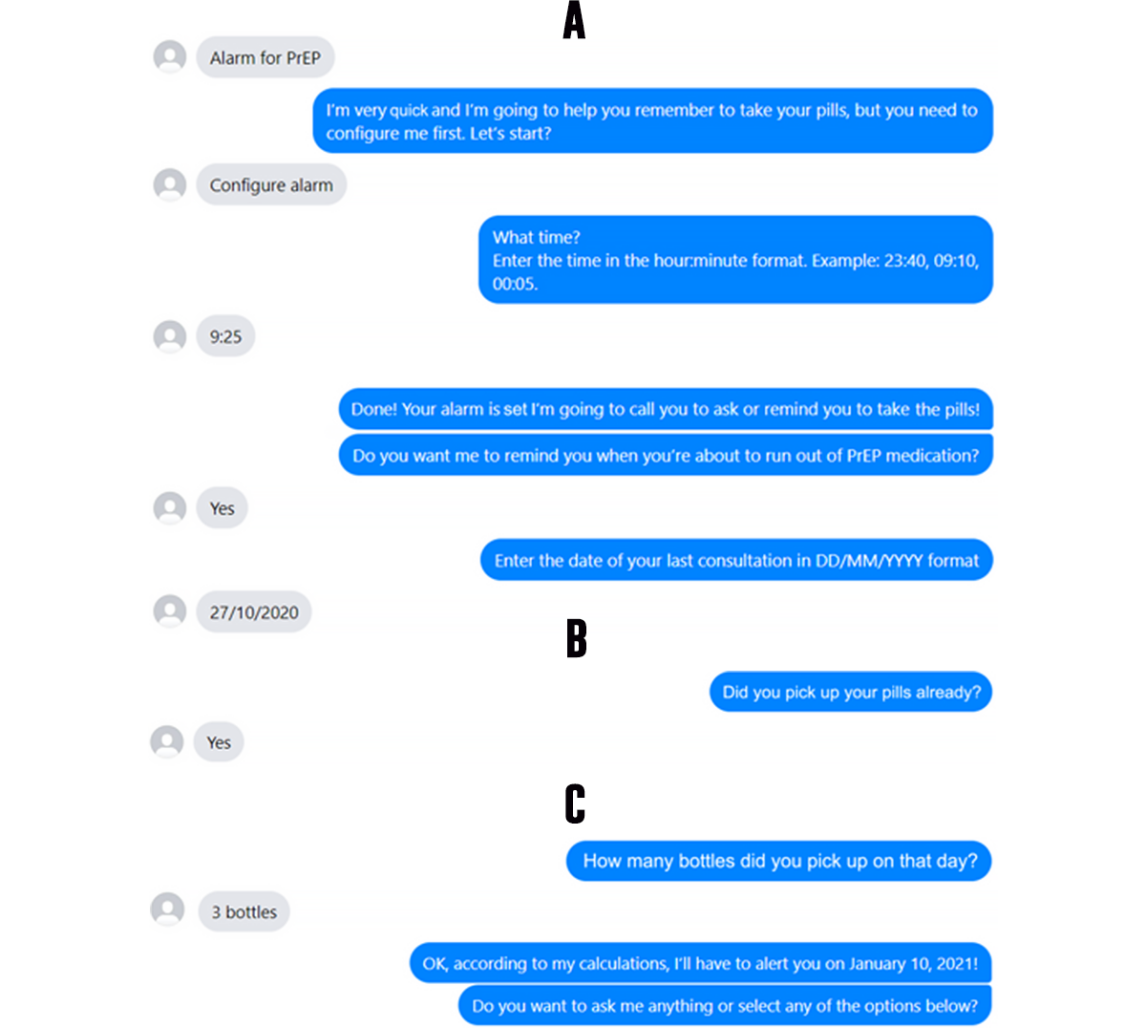
A: Setting a daily reminder for the user to take their pills. B: Amanda’s message to remind the user to take the pills. C: Setting a reminder that the pills are about to run out. PrEP: pre-exposure prophylaxis.

In another menu, “alarms” are offered to send reminders about pill-taking times, the deadline to renew prescriptions, and new appointments scheduled (Figure 5). These reminders are customized for daily PrEP use. A final set of flows allows users to obtain tips on how to deal with PrEP in daily life, addressing topics such as possible situations of violence, loss of medication, and relationships with family or friends. Those not taking PrEP can ask questions about PrEP or any other preventive methods.

After the usability evaluation of this final version, new flows were added, such as the “I’ve suffered violence” button, to allow participants to report violence and receive guidance on how to access health care services; and the “About Amanda Selfie” button, in which the chatbot shares her biography and explains how the experience of sexual violence is part of her persona. Regarding Amanda’s text, revisions were made to balance using abbreviations and slang. Concerning the repertoire of responses in the open flow, continuous training of the chatbot was maintained by introducing new questions and answers in its programming. Additionally, in terms of content, we included images in some balloons to illustrate technical terms related to the prevention of HIV and other STIs.

As a result of usability evaluation, Amanda’s programming underwent some changes to ensure an adequate response time and mitigate the fast pace typical of human-machine interaction. These changes also aimed to guarantee agility and promptness in feedback. The timer was adjusted for individual needs, which determines the number of seconds the chatbot should take to respond to a question or for a button to be selected by the user. This way, longer texts are given longer reading times, and the time stipulated for the appearance of the first balloon was increased from 1 to 3 seconds.

The qualitative evaluation demonstrated the good usability of the chatbot. Users mainly accessed it via a smartphone for an average of 15 minutes. Most scored 9 or 10 out of 10 and reported that they would recommend Amanda Selfie to a friend. Participants valued that she is a TGW and a “well-functioning” chatbot. They used terms such as “intuitive,” “iconic,” “trans,” and “instructive” to define her.

**Figure 6:**
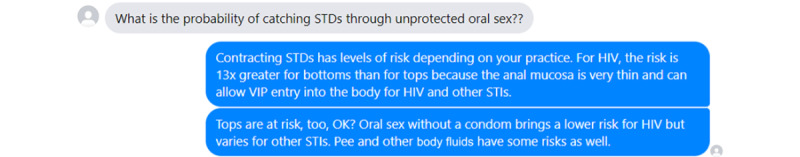
Example of question from a user answered by Amanda. PrEP: pre-exposure prophylaxis; STD: sexually transmitted disease; STI: sexually transmitted infection.

Amanda’s communication was straightforward and easy to understand, mainly because of the introduction of messages with examples and detailed explanations (Figure 6). Most users evaluated the mix of elements of the everyday internet language (ie, abbreviations, memes, and emojis), nonbinary expressions, and slang terms from Pajubá positively. However, 2 participants suggested a “less exaggerated” use of slang, arguing that these linguistic resources could be perceived as grammatical errors.

Adolescents reported feeling comfortable talking to Amanda Selfie about sexuality, emphasizing that they felt safer and less exposed to judgments talking to a chatbot than to humans. However, they also reported the importance of migrating to a dialog with a “real” person on topics that required greater depth, such as doubts about the daily use of PrEP and family problems.

Users pointed out 3 main limitations. The first referred to Amanda Selfie’s inability to interpret some of the questions asked by users in the open flow, meaning that the chatbot did not fully grasp the diversity of the interactions and, consequently, its answers were inaccurate.

The second limitation relates to the time interval between the questions being asked by users and Amanda Selfie’s response. Although Amanda’s agility in answering questions was most appreciated, some respondents observed that being too agile reminded them that they were talking to a robot and did not give them enough time to read all the information provided. Successive balloons also appear too quickly or contain long answers, and users must scroll the screen up and down to read. The third limitation relates to using technical terms, such as blister and wart, when explaining an STI.

The following were suggestions from the interviewees to improve Amanda that were not incorporated (because of the mismatch between required and available time) in the final version: a version of the chatbot for family members of AMSM and ATGW; including videos to explain STI prevention; and using other social networks to expand Amanda’s reach, especially WhatsApp and Instagram.

### Analysis of Amanda’s Result of Demand Creation for PrEP

Amanda Selfie interacted at least once with 757/1239 (61.1%) people who were reached by the DCSSN. This number is higher than what was observed in other demand-creation strategies carried out on social media, such as Instagram (n=332) or Facebook/WhatsApp (n=150; Table 1)

However, Amanda Selfie achieved a lower number of first interactions with MSM and TGW aged 15-19 years in both absolute numbers (n=130) and in the proportion (130/757, 17.2%). Those numbers in the other online social media were 286/332 (86.1%) for Instagram and 73/150 (48.7%) for Facebook/WhatsApp. The difference in the capacity to interact with adolescents was more expressive on Instagram when compared with Amanda (Table 1).

Concerning access to the PrEP clinics through Amanda Selfie, the interactions performed either actively or passively by peer educators on online social media (Instagram, Facebook, and WhatsApp) were more successful. Only 16.2% (21/130) of Amanda Selfie’s first interactions with adolescents resulted in PrEP clinic access, compared with 32.2% (92/286) among those approached on Instagram. Notably, after the interaction with Amanda Selfie, 19 adolescents were enrolled in PrEP, constituting 17.4% (19/109) of the total number of PrEP uptake achieved by DCS on social networks (Table 1).

Regarding the profile of the AMSM/ATGW reached by DCS (Table 2), Amanda Selfie was used (*P*<.001) in a more significant proportion by ATGW (20/130, 15.4%), users with higher education levels (46/130, 35.4%, with complete or incomplete university degree), and White users (68/130, 52.3%).

**Table 1:** Number of initial interactions and users who initiated PrEP^a^ after interacting with Amanda and other social media (initiation of PrEP: June 30, 2019, to March 31, 2021).

Cascade steps	Amanda, n (%) (N=757)	Instagram, n (%) (N=332)	Facebook^b^/WhatsApp, n (%) (N=150)
Interactions with adolescents^c^	130 (17.2)	286 (86.1)	73 (48.7)
Scheduled in PrEP clinics^d^	21 (16.2)	92 (32.2)	39 (53.4)
PrEP uptake^d^	19 (14.6)	59 (20.6)	31 (42.5)

^a^PrEP: pre-exposure prophylaxis.

^b^Other Facebook pages, with the Messenger being answered by a peer educator.

^c^Sexually active homosexual males and transgender females, 15 to 19 years old, at an increased risk for HIV.

^d^Percentage relates to the total number of interactions with adolescents.

**Table 2:** Demographic profile of adolescents reached by demand creation strategies.

Sociodemographic characteristics			Amanda, n (%) (N=130)		Social networks^a^, n (%) (N=359)		Face-to-face, n (%) (N=799)		Total, n (%) (N=1288)		*P* value
**Age (years)**											.28
	15-17	42 (32.3)		113 (31.5)		220 (27.5)		375 (29.1)			
	18-19	88 (67.7)		246 (68.5)		579 (72.5)		973 (75.5)			
**Gender**											<.001
	Men who have sex with men	109 (83.8)		315 (87.7)		684 (85.6)		1108 (86.0)			
	Transgender women	20 (15.4^b^)		18 (5.0)		79 (9.9)		117 (9.1)			
	Nonbinary	1 (0.8)		26 (7.2)		36 (4.5)		63 (4.9)			
**School**											<.001
	Up to elementary school complete	13 (10.0)		49 (13.6)		62 (7.8)		124 (9.6)			
	High school; incomplete/complete	71 (54.6)		217 (60.4)		593 (74.2)		881 (68.4)			
	University; incomplete/complete	46 (35.4^b^)		93 (25.9)		144 (18.0)		283 (22.0)			
**Skin color**											<.001
	Black	24 (18.5)		191 (53.2)		296 (37.0)		511 (39.7)			
	Brown	29 (22.3)		94 (26.2)		251 (31.4)		374 (29.0)			
	White	68 (52.3^b^)		62 (17.3)		222 (27.8)		352 (27.3)			
	Others^c^	9 (6.9)		12 (3.3)		30 (3.8)		51 (4.0)			

^a^WhatsApp, Instagram, and Facebook.

^b^Standardized residue >1.96.

^c^Yellow and Indigenous.

## Discussion

### Principal Findings

The use of a TGW chatbot as a persona to promote access to PrEP among AMSM and ATGW was well accepted and contributed to a slight increase in PrEP uptake, especially among TGW. This finding is important because TGW face more barriers to accessing health care services independently. The use of resources that brought the chatbot closer to the context of AMSM and ATGW, such as the language used on the internet, nonbinary communication, and the creation of more objective flows focused on the primary purpose of the chatbot, contributed to this result. By contrast, Amanda Selfie’s results were more limited than other DCSSNs, which had higher PrEP uptake rates [19].

PrEP coverage globally remains low [25] and strategies that facilitate access to health care services are essential, especially for the more vulnerable population groups. Our results showed that Amanda Selfie has the potential to contribute in that sense, with the chatbot helping one-sixth of the adolescents who contacted her to reach health services.

Two aspects improved these results: more agile and direct flows and combining interactions with the chatbot and humans. Although an initial contact with a chatbot can help overcome adolescents’ apprehension regarding confidentiality of the information and parental consent needs, contact with humans afterward seems to allow for a greater degree of personalization and to provide support for adolescents compared with an intervention based only on artificial intelligence. The contact with a person allows for negotiating the best day and time, the possibility of arranging a meeting point inside or outside the health institution, and provides the support to make the commute safer. Similar results on the benefit of complementary interactions with health professionals and other adolescents were found by Beaudry et al [26], who analyzed a chatbot to engage adolescents in self-care.

Previous research has shown that chatbots can be valuable tools to reduce people’s resistance to addressing topics with a more significant potential for embarrassment [13]. Agreeing with Ho et al [27], participants highlighted feeling safer while disclosing intimate information to a computer as compared with a person, especially when talking about sexual experiences that they felt might be judged by others. The feeling of greater confidentiality described by the participants may be related to the standardization of the virtual approach of a chatbot, that is, there is no variation in the reaction to the information given by the participants, which could be understood as free of judgment, even if their interactions are monitored by humans. Participants having direct contact with humans were not foreseen during the demand creation flow, unless or until they clicked that specific button.

Unlike the findings by Peng et al [14], no references were found in interviews expressing concerns about the risk of lack of confidentiality related to the personal information transmitted to Amanda using the Facebook Platform. It is important to point out that the virtual approach can be felt safer, especially in a research context, in which there is a commitment to not reveal user identity and there are explicit security protocols for storing and using research data. However, as we encourage the provision of sensitive information and we have no governance over the use of data by the platform, nor whether another person could have access to the participant’s electronic equipment used for their virtual interaction, we alerted participants about these possibilities and encouraged the adoption of security measures during their interactions with Amanda.

The main challenge related to the development of Amanda Selfie was to guarantee the full functioning of the closed and open flows, both from an operational and content point of view, because high performance was a condition to create PrEP demand. Amanda Selfie created an opportunity for AMSM and ATGW to talk about sexuality and PrEP without the concern to be judged, using short, direct texts with nonscientific writing and visual resources in a research context. This approach contributed to adolescents’ feelings of comfort when initiating and continuing interactions, leading to the excellent acceptability and usability of the chatbot.

Although the percentage of AMSM and ATGW who contacted the project through the chatbot was considerably lower than those who did so through other online social media, Amanda Selfie contributed to an increase in the number of PrEP uptake events by 2.4% (n=807-826). Some aspects may have limited the chatbot’s efficiency in this regard: being initially linked to only 1 social media platform (ie, Facebook Messenger); being on a new page created for research and prevention purposes, not previously known by the population it aimed to reach; and being passive, depending on the user’s initial contact. These aspects may also suggest why MSM and TGW with higher socioeconomic status and having more access to technology were the main users of Amanda.

Crutzen et al [28] noted the importance of health-related chatbots in addressing a wide range of young people’s needs and interests (eg, questions related to sex, drugs, alcohol). In the present case, including themes related to sexuality within the scope of the chatbot may have been an advantage. Data on sexual experiences, gender identities, and sexual orientation were accessed in more than one-third of the interactions, corroborating the conclusion of Hightow-Weidman [29] on the greater engagement of young people with HIV prevention platforms when they are associated with content that promotes self-acceptance and physical and emotional well-being.

Finally, the choice of a persona representing social minorities, in this case, transgender and Black females, was considered positive by most interviewees, fulfilling the function of generating greater empathy among peers. This greater empathy was reflected in the chatbot’s ability to attract TGW to PrEP. However, critiques regarding a possible exaggeration of the chatbot’s sensuality and language characteristics indicate the need to avoid the reinforcement of stereotypes associated with transgender women carefully.

### Conclusion

Using a chatbot with a persona identified with the most vulnerable populations to HIV can contribute to reducing deficits in PrEP coverage. However, complementary interactions with humans and strategies to disseminate the tool are essential to reduce access inequalities and increase the impact of the chatbot on the population.

## Appendix

Multimedia Appendix 1 Themes included on the database of Amanda´s open flow.

Multimedia Appendix 2 Examples of closed flows to pre-exposure prophylaxis (PrEP) demand creation.

## Abbreviations

AMSM: adolescent men who have sex with men
ATGW: adolescent transgender women
DCS: demand creation strategy
DCSSN: demand creation strategies based on social networks
MSM: men who have sex with men
PrEP: pre-exposure prophylaxis
STI: sexually transmitted infection
TGW: transgender women
