# Supplementary material for: A Transgender Chatbot (Amanda Selfie) to Create Pre-exposure Prophylaxis Demand Among Adolescents in Brazil: Assessment of Acceptability, Functionality, Usability, and Results
Source: J Med Internet Res. 2023 Jun 23;25:e41881. doi: 10.2196/41881 (PMC10337301; doi:10.2196/41881)
Supplement: Multimedia Appendix 1 [file jmir_v25i1e41881_app1.pdf]

69	gouinage	risk of each practice
abuse	cum outside/inside	pink and blue
asexual activity – akuendar	haters	come out of the closet
condom alternative	hepatitis	bareback
blue Amanda	herpes	meaning of life
boring Amanda	hypotenuse	anal sex
Amanda’s age	HIV cure	sex when going out
Amanda doesn't understand	HIV and aids	lesbian sex
Amanda’s topics	HIV and weather	sex on the street
top/bottom	HPV	crush sexuality
self-test	gender identity	syphilis
Greek kiss	undetectable	silicone Amanda
STI from kissing	start PrEP	HIV/STI symptoms
enema pump	intersex	about condoms
sex toys	common STIs	about pubic lice
Amanda’s pick-up	send nudes	about gender
serodiscordant couples	masturbation and orgasm	about gonorrhea
cis and trans	suck me	about live broadcasts
Chlamydia	medication	about non-binarity
guinea pig / mistrust	fear of being tested	about the alarm
withdrawal	NB likes NB	about sexual orientation
how are you	contraceptive methods	about pep
secret conversation	gender reassignment	about PrEP
goodbyes	Amanda’s sex change	about oral sex
difference between drag and trans	objective of the research	about testing
difference between PrEp and PEP	where to find PrEP	I am beautiful
difference between sex and gender	what is a woman?	am I LGBT?
difference between transsexual and transvestite	what are STIs	it's raining
doubts about PrEP among PrEP users	what do you like?	time with a condom
condom effectiveness	pansexual/bisexual	I have/I’ve been diagnosed with HIV
PrEP effectiveness	flabbergasted!	testing
compliments	piercing and sex	transsexuality school
find a doctor	Pompoir	trichomoniasis
search addresses	PrEP and drugs	violence
LGBT places	PrEP and prejudice	I’m a virgin, what do I do
fetishes	PrEP and antiretrovirals	voucher
phimosis HIV	PrEP injectable	voyeur
flirt	occasional PrEP	swear words
genderfuck	problems with parents	anal douche
golden shower	PrEP at what address?	
