# Supplementary material for: A Transgender Chatbot (Amanda Selfie) to Create Pre-exposure Prophylaxis Demand Among Adolescents in Brazil: Assessment of Acceptability, Functionality, Usability, and Results
Source: J Med Internet Res. 2023 Jun 23;25:e41881. doi: 10.2196/41881 (PMC10337301; doi:10.2196/41881)

Multimedia Appendix 2: Examples of closed flows to PrEP demand creation

Caption

→ Arrow: indicates flow direction

Rectangle: Amanda's answer buttons or speech bubbles

Diamond: decision-making points between differents paths

Square: with double side borders connected to the dotted line: guidance for classifying users

INITIAL FLOW + POPULATION OF INTEREST

The initial flow comprises welcome messages as well as the Target Group Quiz.

To be identified as targert group, users must answer questions **A1, A2, A3, A6 and A6a** with specific answers corresponding to the study's criteria. When they do so, the chatbot offers them to participate in a survey, and they will fill out the recruitment form.

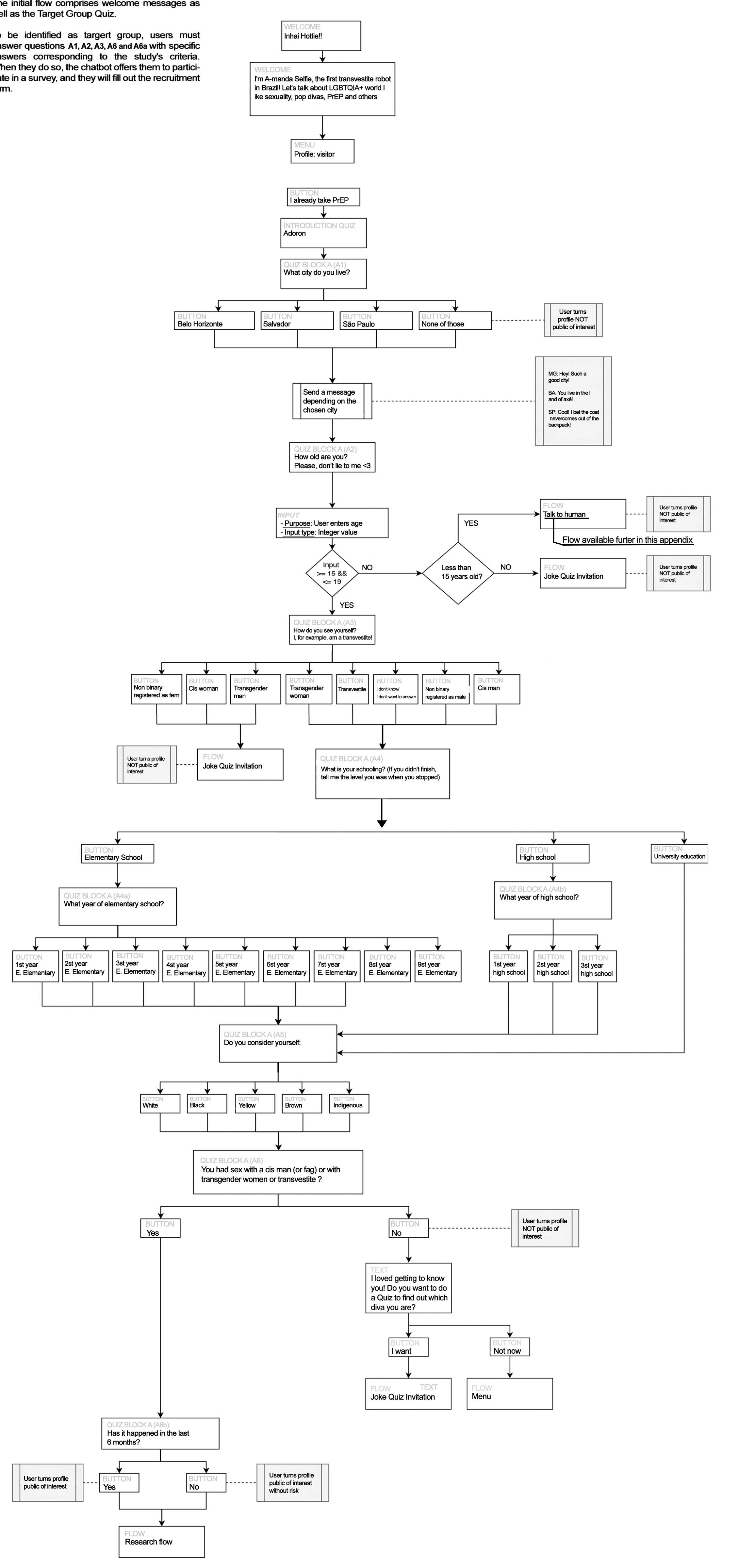

DOUBTS (NON-PrEP) FLOW

The Doubts (Non-PrEP) Flow has the objective of resolving sensitive doubts about PrEP for non-PrEP users.

After removing the doubt (message), the user will be directed to make an appointment to consult with the PrEP team (scheduling flow).

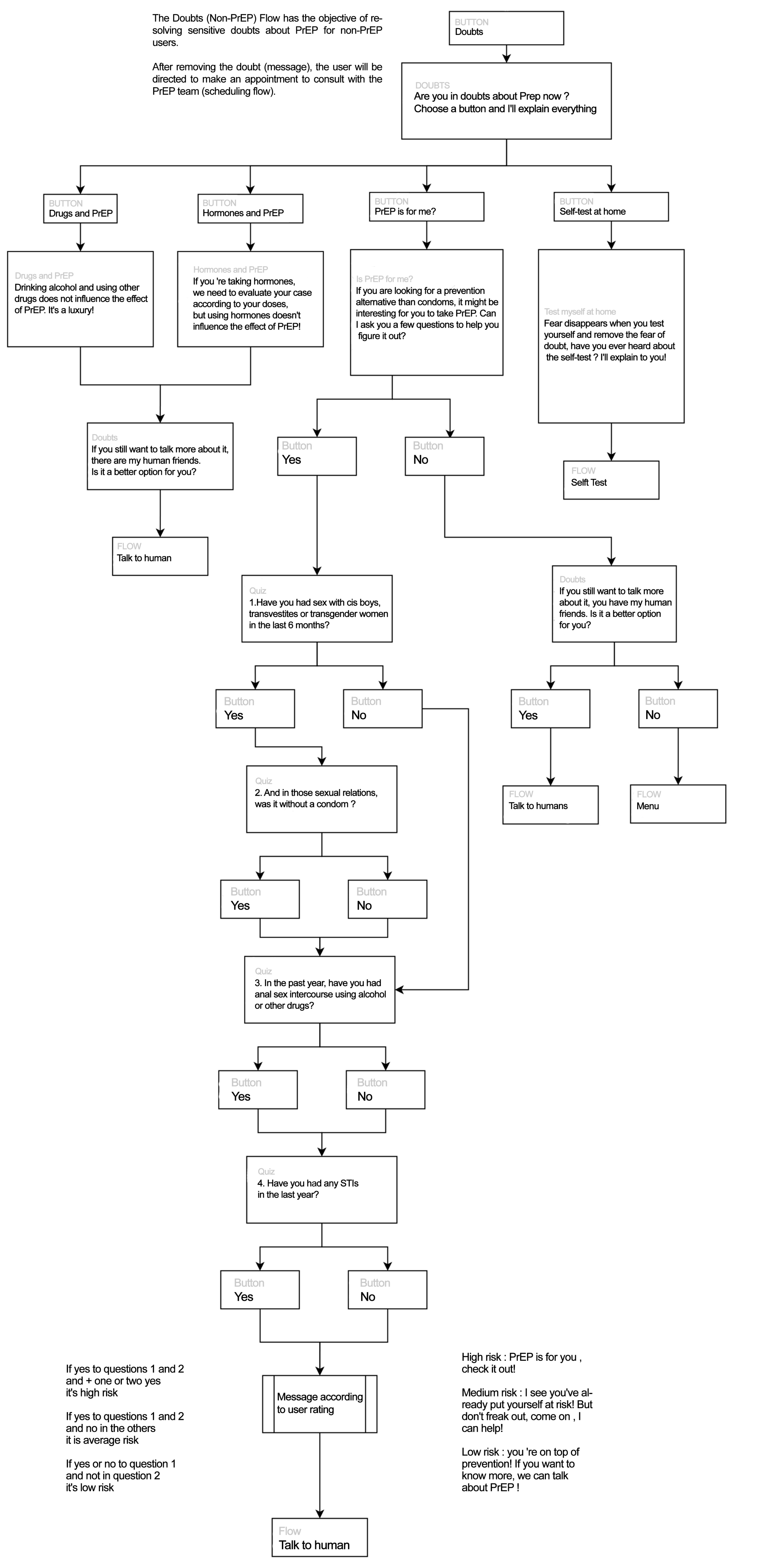

TALK TO HUMAN FLOW

This flow has the objective of presenting options for the user to get in touch with a human from one of the programs

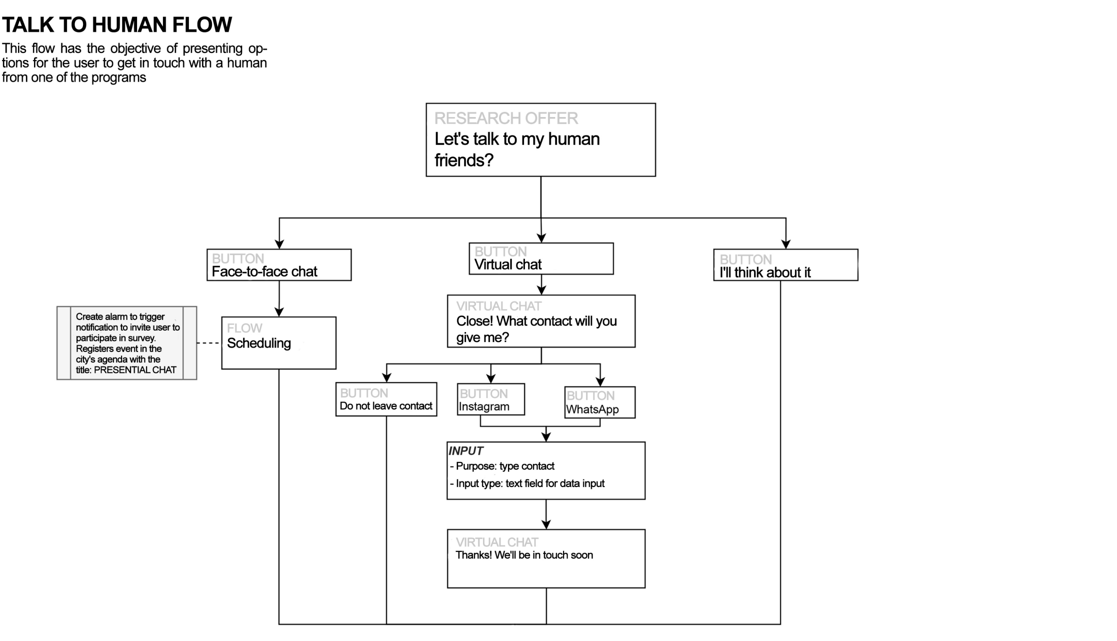

SELF-TEST FLOW

This flow offers testing options to the user. The options depend on availability in each city.

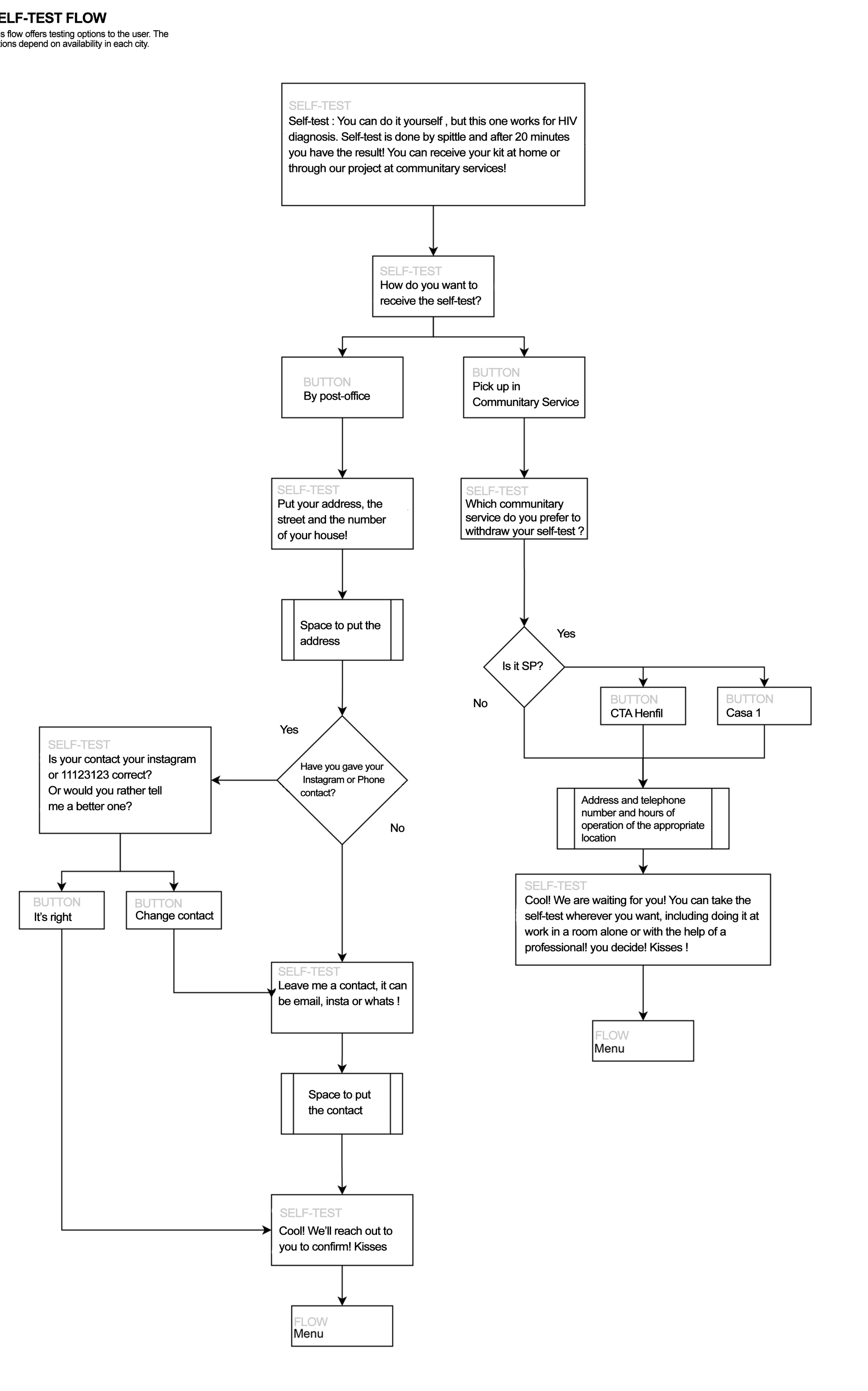

Supplement: Multimedia Appendix 2 [file jmir_v25i1e41881_app2.pdf]
